# Mortality Rates of Surgical Techniques for Correcting Atrioventricular Disjunction

**DOI:** 10.21470/1678-9741-2020-0041

**Published:** 2020

**Authors:** Élcio Pires Junior, Marcelo Luiz Peixoto Sobral

**Affiliations:** 1 Hospital Beneficência Portuguesa de São Paulo, São Paulo, Brazil.

**Keywords:** Mitral Valve, Heart Ventricles, Papillary Muscles, Pericardium, Heart Atria, Pericardium, Tricuspid Valve

## Abstract

We compared the mortality rates of two surgical techniques for correction of atrioventricular disjunction in 10 out of 720 patients who underwent mitral valve replacement from 2005 to 2012. In group I, the mitral annulus was fixed with bovine pericardial strips; in group II, a 'patch' of bovine pericardium was sutured and extended from the base of the lateral and medial papillary muscles, covered the posterior wall of the left ventricle, went through the posterior mitral annulus, and ended in the posterior wall of the left atrium adjacent to the mitral ring. The group II technique showed a lower mortality.

**Table:** 

Abbreviations, acronyms & symbols	
**LVR**	**= Left ventricular rupture**
**MVR**	**= Mitral valve replacement**

## INTRODUCTION

Atrioventricular disjunction associated with left ventricular rupture (LVR) is a rare and serious complication following mitral valve replacement (MVR)^[1]^. It was initially described by Roberts and Morrow, in 1967, from autopsy findings in two patients^[2]^. In 2019, Van shared his experience with MVR in 744 patients, and in a 30-day postoperative period, LVR has occurred in one patient^[3]^.

The etiology of LVR includes the replacement of a previously implanted prosthesis, selecting a larger prosthesis than the mitral annulus and the left ventricle, weakness of the left ventricular wall due to cardiomyopathy, mechanical traction injuries of the mitral annulus, and excessive papillary resection, while the key factors are mitral stenosis and calcification of the mitral annulus^[4]^. However, in most patients, the specific cause cannot be determined^[5]^.

Consequently, a safe and reproducible surgical technique is necessary for dealing with this complication. In this study, we compared the mortality rates of two surgical techniques for the correction of LVR.

## TECHNIQUE

### Methods

A retrospective study was carried out using data collected from January 2005 to January 2012, during which time 720 surgeries involving MVR were performed. Valve prostheses (biological or mechanical) were used for MVR as the primary surgery. MVR associated with other procedures (myocardial revascularization, multiple valve replacement, aortic surgery, etc.) and MVR during reoperation were excluded of this study.

All procedures were performed under extracorporeal circulation, with arterial cannulation in the ascending aorta and bicaval venous cannulation with moderate hypothermia (28 °C). The myocardial protection was performed by cold blood cardioplegia (4:1 dilution) every 20 minutes. The mitral valve was accessed by left atriotomy.

**Fig. 1:**
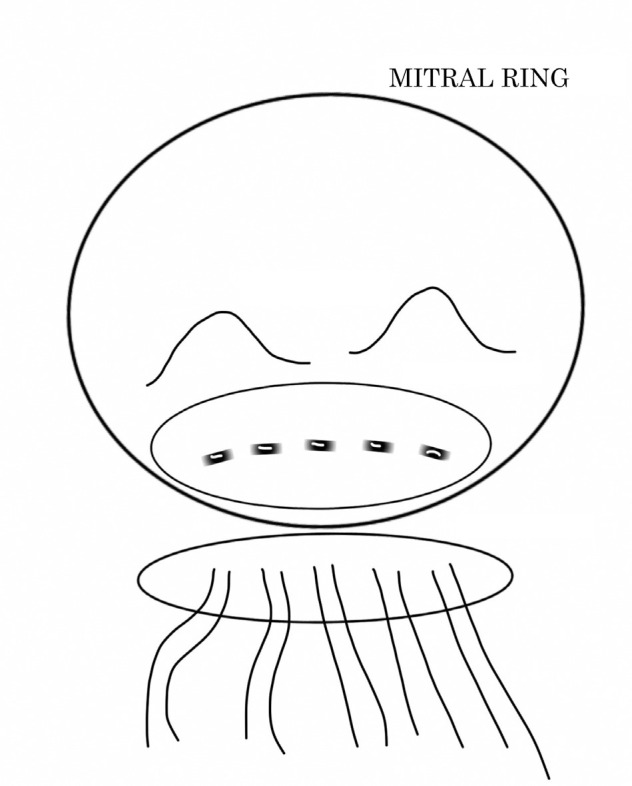
Schematic drawing of the surgical technique performed in group I. Establishment and strengthening of the posterior mitral ring with two strips of bovine pericardium and separate polypropylene 3-0 "U" sutures in the ruptured region, using the same sutures to implant the prosthesis.

**Fig. 2:**
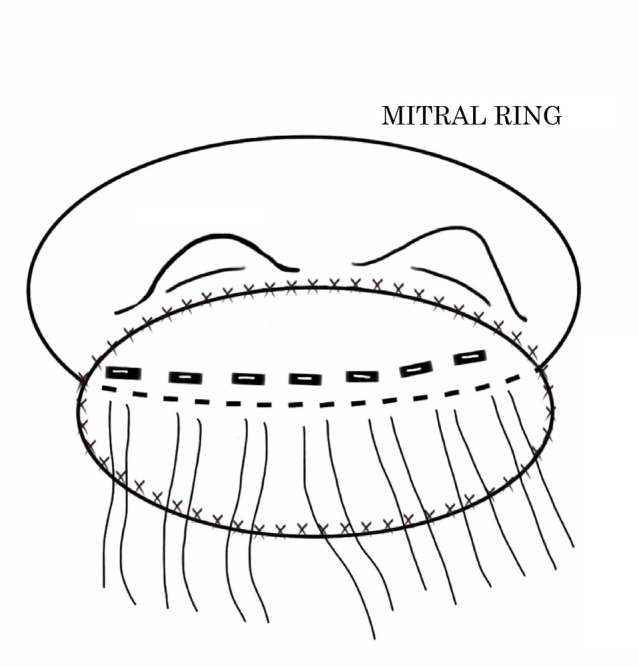
Schematic drawing of the surgical technique performed in group II. Placement of a bovine pericardial patch, fixed with continuous polypropylene 4-0 sutures and extending from the base of the medial and lateral papillary muscles, covering the posterior wall of the left ventricle through the posterior portion of the mitral valve annulus and ending in the posterior wall of the left atrium adjacent to the mitral annulus. The prosthesis was placed on this patch and separate "U" sutures supported the bovine pericardium allowing the correction of external hemorrhages.

Following these surgeries, LVR was observed in 10 patients who were further divided into the following two groups according to the type of corrective technique adopted: group I (n=6), which involved the establishment and strengthening of the posterior mitral ring with two strips of bovine pericardium and separate polypropylene 3-0 "U" sutures in the ruptured region, using the same sutures to implant the prosthesis, securing the location of the lesion, and using biological adhesive and bovine pericardium to correct external hemorrhages (Figure 1); and group II (n=4), which involved placement of a bovine pericardium patch, fixed with continuous polypropylene 4-0 sutures and extending from the base of the medial and lateral papillary muscles, covering the posterior wall of the left ventricle through the posterior portion of the mitral valve annulus, and ending in the posterior wall of the left atrium adjacent to the mitral annulus. The prosthesis was placed on this patch and separate "U" sutures supported the bovine pericardium allowing the correction of external hemorrhages (Figure 2). These surgeries occurred in sequential way.

The LVR was classified into three types ranging from its position in relation to the mitral annulus and papillary muscle: Type I, the LVR is located in the mitral annulus and adjacencies; Type II, the rupture occurs at the base of papillary muscles; and Type III, the tear is mid-ventricular and located in the region between the mitral annulus and the papillary muscles.

There was no need for replacement of the prosthesis in any of the groups, and all prostheses were biological and manufactured from bovine pericardium. The project was approved by the Scientific Ethics Committee of the Real e Benémerita Associação Portuguesa de Beneficência de São Paulo (opinion 209.339).

### Statistical Analysis

The statistical analysis of all data collected from this survey initially included descriptive means. For quantitative (numerical) variables, some summary measures, such as mean and standard deviation, were calculated, and one-dimensional scatterplots were drawn. Qualitative (categorical) variables were analyzed through the calculation of absolute and relative frequencies (percentage) in addition to the construction of bar graphs.

Inferential analyses were employed in order to confirm or refute evidence found in the descriptive analysis as follows: Fisher's exact test or its extension for the comparison of groups I and II according to sex, mitral valve disease, calcification of mitral annulus, LVR, and death^[6]^; Student's *t*-test for independent samples for the comparison of groups I and II according to the mean age, left atrial size, and ejection fraction value before surgery^[7]^; Mann-Whitney test for the comparison of groups I and II according to hospitalization time^[8]^.

A significance level (α) of 5% was used in all the inferential analyses. The data were entered in a Windows Excel 2010 spreadsheet for proper storage of information. Statistical analyses were performed with the IBM Corp. Released 2010. IBM SPSS Statistics for Windows, Version 19.0. Armonk, NY: IBM Corp.

## RESULTS

The sample for this study consisted of 10 patients with LVR after MVR. In six patients, LVR was corrected by fixing the mitral annulus with strips of bovine pericardium (group I) and in the remainder, the suture technique for an extra-annular bovine pericardium patch was used (group II).

Group I included five (83.3%) women and one (16.7%) man and group II included three (75.0%) women and one (25.0%) man (*P*=0.19). The average age of patients in group I was 59.8 years (range from 53 to 66 years) with a standard deviation of 5.4 years, and the average age of patients in group II was 53.8 years (range from 40 to 72 years) with a standard deviation of 15.0 years (*P*=0.49). Among the mitral valve disease patients in group I, four (66.7%) patients had mitral insufficiency and stenosis (stenosis > insufficiency) and two (33.3%) presented with pure mitral stenosis. In group II, one (25.0%) patient had mitral insufficiency and stenosis (stenosis > insufficiency), one (25.0%) had mitral stenosis, and two (50.0%) had mitral insufficiency (*P*=0.19). All patients from group II presented with calcification of the mitral annulus.

In group I, before surgery, the average left atrial size was 57.8 mm (range from 46 to 74 mm) with a standard deviation of 10.6 mm. After surgery, the left atrial size in the only survivor was 62 mm. The mean ejection fraction value before surgery was 69.0% (range from 63 to 79%) with a standard deviation of 5.8%, and after surgery, the ejection fraction value was 48.0%. In group II, before surgery, the average left atrial size was 51.5 mm (range from 44 to 56 mm) with a standard deviation of 5.3 mm, and after surgery, the average left atrial size was 55 mm (range from 49 to 59 mm) with a standard deviation of 4.5 mm. The mean ejection fraction value before surgery was 67.8% (range from 50 to 80%) with a standard deviation of 12.7%, and after surgery, the mean ejection fraction value was 57.8% (range from 45 to 71%) with a standard deviation of 14.2%.

All six (100%) patients in group I were hospitalized for 11.3 days (range from one to 63 days) with a standard deviation of 25.3 days. In group II, the patients were in hospital for 26.3 days (range from nine to 54 days) with a standard deviation of 20.4 days (*P*=0.11).

In group I, five patients died in the surgical center and one had late death. In group II, only one patient died (late death) (*P*=0.01). Others details of the groups’ profiles are presented in Tables 1 and 2.

**Table 1:** Distribution of the groups' profiles according to left ventricular rupture.

Left ventricular rupture	Group I	Group II	Total	*P*-value
I	6 (100%)	2 (50%)	8 (80%)	0.13*
III	-	2 (50%)	2 (20%)	
Total	6 (100%)	4 (100%)	10 (100%)	

* Fisher's exact test or its extension.

**Table 2:** Distribution of the groups' profiles according to the left atrial size and ejection fraction value before and after surgery.

	Group I	Group II	*P*-value
Left atrium (mm)			0.31*
Before surgery	57.8±10.6	51.5±5.3	
After surgery	62±0.5	55±4.5	
Ejection fraction (%)			0.84*
Before surgery	69±5.8	67.8±12.7	
After surgery	48±11.8	57.8±14.2	

* Student's t-test for independent samples.

The inferential results from the comparison of groups I and II showed that these profiles are statistically similar for sex (*P*=0.19), age (*P*=0.49), mitral valve disease (*P*=0.19), LVR (*P*=0.13), hospitalization time (*P*=0.11), left atrial size (*P*=0.31), and ejection fraction value (*P*=0.84). The comparison of the left atrial size and ejection fraction value before and after surgery can only be carried out in a descriptive manner due to the small number of patients who did not die during surgery in both groups.

The only patient in group I that did not die in surgery showed a decrease in both the left atrial size (74 mm to 62 mm) and the ejection fraction value (63% to 48%). In group II, the four patients who did not die in the operation theater showed an increase in the left atrial size and a decrease in ejection fraction value. Considering only the deaths (both in the operation theater and the late deaths), group I had six deaths, and group II only one.

In group I, the five deaths were of four women and one man. The average age of the individuals who died was 59.8 years (range from 53 to 66 years) with a standard deviation of 5.4 years. In terms of mitral valve diseases, four deaths were because of mitral insufficiency and stenosis (stenosis > insufficiency) and two were caused by mitral stenosis. All six patients from group I who died showed calcification of the mitral annulus and Type I LVR. On average, the patients in this group were in hospital for 11.3 days (range 1 to 63 days) with a standard deviation of 25.3 days. The only death in group II was of a 72-year-old woman with mitral insufficiency and Type III LVR, and she remained in the hospital for nine days. In seven cases (70%), the posterior leaflet and its subvalvular apparatus were preserved.

From group I, six patients (100%) died, with five deaths (83.3%) in the operation room and one late death (16.6%) from pulmonary sepsis. In group II, there was one late death (25%), which was associated with pulmonary sepsis in the late postoperative period.

When we analyzed the postoperative period, the mean length of hospital stay was 39 days and there was a 7% decrease in the ejection fraction value on average, similar to that found in presurgical transthoracic echocardiography. There was no evidence of myocardial infarction; electrocardiograms showed no ischemic changes and cardiac enzymes remained within the standards expected for surgical cardiac procedures.

## DISCUSSION

The surgical treatment of LVR presents a series of difficulties, such as weakness in the structures causing difficulty in anchoring sutures, poor exposure and problematic visualization of lesions, proximity of lesions to the circumflex coronary artery, and perforation of the left ventricular wall further from the initial lesion^[9]^. In both groups, the patients progressed to a cardiogenic shock with great difficulty in completion of the repair, regardless of the technique used. In all cases, successful or otherwise, the LVR was evidenced by hemorrhage in the free left ventricular wall.

In the cases examined, a greater occurrence of LVR Type I was observed, indicating that the probable excessive manipulation of the mitral annulus and calcification were responsible for those complications. And we remind that LVR is classified into three types ranging^[10-12]^.

LVR can be avoided with the maintenance of the posterior apparatus of the partial or complete mitral valve. In cases where there is extensive valve calcification, removal of calcium should be sufficient only for the passage of the sutures through the ring and fixation of the prosthesis, avoiding hypertension in the postoperative period^[5,12]^. Although the literature shows a reduction in the occurrence of LVR during the preservation of the posterior apparatus of the mitral valve, in our sample, the posterior apparatus was maintained in most cases.

A high mortality rate is reported in the literature and ranges from 65% to 100%, while survival is associated with its intraoperative identification^[10,13]^. Of the patients included in this study, 10 occurrences of LVR were observed, with a frequency of 1.39%. An overall mortality rate of 60% was observed (100% in group I and 25% in group II). Deaths could be late and without direct correlation with the pathology.

Surgical techniques have been described to correct LVR, based on the healing and strengthening of the tear region, and intraventricular with the use of extracorporeal circulation^[4,10,11,13]^. Patients in group I underwent the type of correction typically used by our service, with interposition of strips of bovine pericardium in the mitral annulus. The technique used in group II began with the extensive suturing of a pericardial patch covering the torn area and the mitral annulus in a random manner, and because of the results, this was retained as the default technique.

There are descriptions of the use of epicardial repair, at the torn ventricular face, without the use of extracorporeal circulation, which is based on the use of biological adhesives to seal the tear^[14]^. No repair was carried out with the use of biological adhesives in the cases described, but they may be useful to minimize the use of sutures that can generate larger lesions in very damaged and fragile tissues.

Observing the literature, we found a higher incidence in patients above 60 years of age, and the calcification of the mitral annulus was considered the main risk factor^[1]^, which was consistent with the one found in the sample studied, with a mean age of 57 years and a predominant pathology of mitral stenosis.

### Limitations

This study has some limitations because it is a retrospective analysis of low incidence pathology, thereby including a low number of study individuals. There was no randomization of surgical techniques analyzed. The procedures were selected according to the experience of the surgeon at the time of surgery.

## CONCLUSION

In the cases analyzed, better results were obtained in terms of mortality with correction of LVR using an extra-ring bovine pericardium patch.

**Table:** 

Authors' roles & responsibilities	
EPJ	Substantial contributions to the conception or design of the work; or the acquisition, analysis, or interpretation of data for the work; drafting the work or revising it critically for important intellectual content; final approval of the version to be published
MLPS	Substantial contributions to the conception or design of the work; or the acquisition, analysis, or interpretation of data for the work; drafting the work or revising it critically for important intellectual content; final approval of the version to be published
